# Microevolution of Nematode miRNAs Reveals Diverse Modes of Selection

**DOI:** 10.1093/gbe/evu239

**Published:** 2014-10-28

**Authors:** Richard Jovelin, Asher D. Cutter

**Affiliations:** Department of Ecology and Evolutionary Biology, University of Toronto, Ontario, Canada

**Keywords:** miRNA, *Caenorhabditis*, gene expression, regulatory networks, nucleotide variation

## Abstract

Micro-RNA (miRNA) genes encode abundant small regulatory RNAs that play key roles during development and in homeostasis by fine tuning and buffering gene expression. This layer of regulatory control over transcriptional networks is preserved by selection across deep evolutionary time, yet selection pressures on individual miRNA genes in contemporary populations remain poorly characterized in any organism. Here, we quantify nucleotide variability for 129 miRNAs in the genome of the nematode *Caenorhabditis remanei* to understand the microevolution of this important class of regulatory genes. Our analysis of three population samples and *C. remanei*’s sister species revealed ongoing natural selection that constrains evolution of all sequence domains within miRNA hairpins. We also show that new miRNAs evolve faster than older miRNAs but that selection nevertheless favors their persistence. Despite the ongoing importance of purging of new mutations, we discover a trove of >400 natural miRNA sequence variants that include single nucleotide polymorphisms in seed motifs, indels that ablate miRNA functional domains, and origination of new miRNAs by duplication. Moreover, we demonstrate substantial nucleotide divergence of pre-miRNA hairpin alleles between populations and sister species. These findings from the first global survey of miRNA microevolution in *Caenorhabditis* support the idea that changes in gene expression, mediated through divergence in miRNA regulation, can contribute to phenotypic novelty and adaptation to specific environments in the present day as well as the distant past.

## Introduction

Intense regulation of genomes by small RNAs forms one of the major biological discoveries of the past decade. The micro-RNAs (miRNAs) comprise one class of small RNAs that play key roles in regulating development and physiology by acting as global managers of gene expression (Ambros 2004; Bartel 2004; Plasterk 2006). The function of miRNAs in regulating tissue identity arose early in animal evolutionary history (Grimson et al. 2008; Christodoulou et al. 2010), with accelerated acquisition of novel miRNA families during the phase of morphological innovation in early vertebrates and during the diversification of mammals and hominoids, supporting the view that miRNAs function to fine tune and buffer expression to facilitate phenotypic diversification (Heimberg et al. 2008; Iwama et al. 2013; Meunier et al. 2013). Moreover, because a single miRNA may regulate hundreds of distinct target messenger RNA (mRNA) gene transcripts (Farh et al. 2005; Lewis et al. 2005), miRNAs exert widespread influence on both genome regulation and genome evolution by imposing selective constraints on the evolution of the 3′ untranslated region (UTR) and protein-coding sequences of their gene targets (Farh et al. 2005; Stark et al. 2005; Cheng et al. 2009; Chen et al. 2011; Jan et al. 2011; Takuno and Innan 2011).

miRNA family composition and homologous relationships in genomes of related species provide a baseline of insights about miRNA formation and evolution (Liu, Okamura, et al. 2008; Berezikov 2011). Even closely related species contain unique sets of miRNA families, indicating that miRNA birth is ongoing and can be rapid, with attendant divergence in miRNA-mediated regulatory networks (Berezikov et al. 2006; Lu, Shen, et al. 2008; de Wit et al. 2009; Fahlgren et al. 2010; Shi et al. 2013). Differential expression of conserved miRNAs could also contribute to phenotypic diversity. For instance, miRNA expression level differences between human populations might cause corresponding variation among individuals in target mRNA expression and drug sensitivity (Huang et al. 2011). Similarly, expression variation in a miRNA causes differences in trichome numbers in *Drosophila melanogaster* (Arif et al. 2013) and disparities in spatio-temporal expression profiles of homologous miRNAs are more pronounced between species that are more distantly related and physiologically dissimilar (Ason et al. 2006).

Despite the clear biological importance of miRNA regulation, the microevolution of miRNA genes is not well understood. Surveys of miRNA sequence evolution have shown that most miRNAs experience intense purifying selection against new mutations, presumably to maintain their regulatory control over gene expression (Saunders et al. 2007; Ehrenreich and Purugganan 2008; Quach et al. 2009; Nozawa et al. 2010; Jovelin and Cutter 2011; Jovelin 2013; Mohammed et al. 2013). In humans, only 65 single nucleotide polymorphisms (SNPs) were retrieved from 474 miRNAs in the SNP database (Saunders et al. 2007), but perhaps twice as many allelic variants per miRNA can be found from direct sequencing of miRNA regions in human populations (Quach et al. 2009). Nevertheless, some miRNA genes evolve without such strong constraint (Liang and Li 2009; Nozawa et al. 2010), consistent with reports of high miRNA gene turnover in mammals and flies (Lu, Shen, et al. 2008; Meunier et al. 2013). Underlining the potential role of miRNA sequence evolution in phenotypic innovation, positive selection for sequence change could be detected on some miRNAs (Lu, Fu, et al. 2008; Quach et al. 2009). The phylogenetic age and expression of miRNA genes correlate inversely with their rate of evolution, with greater sequence conservation for miRNAs of more ancient origin and higher expression level (Ruby et al. 2007; Lu, Shen, et al. 2008; Liang and Li 2009; Fahlgren et al. 2010; Nozawa et al. 2010; Lenz et al. 2011; Shen et al. 2011; Nozawa et al. 2012; Jovelin 2013; Meunier et al. 2013).

The ways in which we should expect natural selection to drive miRNA sequence change on microevolutionary timescales within populations depend critically on the mechanisms of miRNA biogenesis and functional activity. The most important functional product, the ∼22-bp long mature miRNA, is produced through sequential cleavage, first of the primary transcript and then of the stem-loop structured pre-miRNA, respectively, by RNAse III Drosha and Dicer. However, a single pre-miRNA hairpin may generate up to three RNA regulatory species: 1) The mature miRNA (miR), 2) its complementary sequence from the other arm of the miRNA hairpin—the miR*, and 3) loop miRNAs (Okamura et al. 2008, 2013; Yang et al. 2011; Winter et al. 2013). In addition to the potential regulatory complexity afforded by these distinct components, processed variants of the same miRNA gene, called isomiRs, can generate functional diversity (Neilsen et al. 2012). Mature miRs are then loaded in the RNA-induced silencing complex to repress translation of specific mRNA targets through perfect base complementarity of the 5′ miRNA seed motif with the 3′ target UTR, with possible additional target recognition specificity contributed by central sites and 3′ compensatory sites (Bartel 2009; Shin et al. 2010). In addition, Drosha-independent and Dicer-independent pathways also generate miRNAs and pre-miRNA stages could also have a direct role in target recognition (Yang and Lai 2011; Chen 2013). Consequently, the entire hairpin, and especially the mature miR, will be subject to strong purifying selection for both functional activity and proper miRNA processing. Indeed, naturally occurring SNPs outside the mature miR sequence can alter processing and lead to misregulation (Duan et al. 2007; de Meaux et al. 2008; Jazdzewski et al. 2008, 2009). Thus, natural sequence variability in both the miR and its flanking sequence that comprises the pre-miRNA hairpin has the potential to modulate expression of miRNAs and their target genes. It is therefore essential to investigate sequence evolution of the entire stem-loop hairpin to determine how evolution of miRNAs can alter regulatory networks.

The nematode *Caenorhabditis remanei* provides a powerful system to study microevolutionary change. The genome of *C. remanei* contains an exceptionally high density of SNPs in natural populations, with ∼4% of nucleotide sites expected to differ between a pair of alleles in the absence of selective constraint (Cutter et al. 2013). The short genetic distance at unconstrained sites between *C. remanei* and its sister species *Caenorhabditis latens*, formerly known as *C*. sp. 23 (Dey et al. 2012; Felix et al. 2014), makes interspecies comparisons useful for inferring the directionality of mutational changes from their common ancestor and for detecting adaptive evolution (Jovelin et al. 2014). *Caenorhabditis elegans* also is a close relative and the species in which miRNAs were first discovered (Lee et al. 1993), and so miRNA content in *C. remanei* has been investigated through RNA-seq (de Wit et al. 2009; Shi et al. 2013). Previous analyses of miRNA evolution in *Caenorhabditis briggsae* and *Caenorhabditis nigoni* (formerly known as *C.* sp. 9) identified changes in miRNAs predicted to alter gene regulation but were based on interspecies divergence or allelic variation in a limited number of miRNAs (Jovelin and Cutter 2011; Jovelin 2013). Consequently, the *C. remanei* genome presents a particularly powerful system in which to quantify the microevolutionary pressures on miRNAs on a per-gene basis, side stepping many challenges of studying per-gene evolution in natural populations of *C. elegans* (Cutter et al. 2009; Rockman and Kruglyak 2009; Andersen et al. 2012) and many other organisms. Here, we take advantage of these virtues of *C. remanei* to quantify miRNA sequence change for most of the miRNAs in the *C. remanei* genome. Our analysis of >400 nt changes in 129 miRNA genes, allelic variation from 3 *C. remanei* populations and interspecies comparisons, identifies population-specific alleles, variants predicted to affect miRNA-induced gene regulation and miRNAs with nonneutral patterns of nucleotide variation despite a predominant signal of ongoing purifying selection against new mutations in contemporary populations.

## Materials and Methods

### Strains

We analyzed *C. remanei* strains originating from three different populations (Dayton, OH; Kiel, Germany; King City, ON) (Cutter 2008; Jovelin et al. 2009; Dey et al. 2012), as well as strains of sister species *C. latens* isolated from Wuhan City, China (Dey et al. 2012). *Caenorhabditis latens* was formerly known as *C*. sp. 23 (Felix et al. 2014). We included four additional strains of *C. remanei* (Japan; Tennessee, United States) and one of *C. latens* (JU724 from Zhouzhuang City, China) for analysis of the *mir-64* cluster. All strains are isofemale lines derived from individuals collected from isopods or decaying vegetal matter, subsequently reared on agar plates seeded with *Escherichia coli* OP50 following standard protocols (Brenner 1974).

### miRNA Data Set

We attempted to analyze population genetic variation for all 149 miRNAs that were first identified in *C. remanei* by RNA-seq (de Wit et al. 2009), but failed to amplify and sequence 25 of them, even after designing a second set of primers. A recent study identified 47 additional miRNAs in *C. remanei* (Shi et al. 2013), and our final data set included 129 miRNA genes. All 129 miRNAs were assessed for allelic variability in a sample from the Ohio population (median = 10 strains per locus), which represents 83% of the miRNAs identified by de Wit et al. (2009) and 69% of the *C. remanei* miRNAs annotated in miRBase v20 (Kozomara and Griffiths-Jones 2011). We analyzed sequence variability for 38 miRNAs in strains sampled from Germany (median *n* = 10 per locus) and Ontario (median *n* = 9 per locus). In addition, we sequenced 79 of the miRNAs in *C. latens*, and collected sequence data from 2 *C*. *latens* strains for a subset of 65 miRNAs.

We included in our data set 20 miRNAs that were identified by de Wit et al. (2009) but that were not annotated in miRBase v20 (*n* = 20 for diversity within Ohio, *n* = 9 for interspecies divergence) because we sought to contrast the evolution of lineage-restricted versus phylogenetically preserved miRNAs. We note, however, that Shi et al. (2013) independently identified 23% of the 40 *C. remanei* candidate miRNAs from de Wit et al. (2009), which are included in miRBase v20. Because of this flux in miRNA recognition, we refer to miRNAs by their name when they are present in miRBase (e.g., *lin-4*, *let-7*, *mir-1*) and use previous authors’ nomenclature for miRNA names when they are absent from miRBase (e.g., block453) (de Wit et al. 2009). We use the notation *mir-1* to refer to the *mir-1* miRNA gene and its hairpin sequence, and miR-1 to refer to its mature sequence.

To test the robustness of our results against possible miRNA misannotation, we repeated our analyses after excluding miRNAs with unusual patterns of polymorphism. Specifically, we removed 21 miRNAs with greater nucleotide diversity in the hairpin region than in non-miRNA flanking sites (12 miRBase-annotated miRNAs: *mir-35b*, *mir-35c-2*, *mir-57*, *mir-59*, *mir-72*, *mir-75*, *mir-236*, *mir-238*, *mir-244*, *mir249*, *mir-252*, *mir-253*; 9 non-miRBase miRNAs: block563, block1224, block1363, block2005, block2830, block2890, block2981, block3884, block4362). Note that this procedure is conservative because it may eliminate bona fide miRNAs that evolve adaptively (Lyu et al. 2014). It is also more conservative than comparing hairpin diversity with synonymous site diversity in protein-coding genes because only five miRNAs have more polymorphism than synonymous sites. We could not compare diversity between flanking sequences and miRNA hairpins for members of the *mir-54* and *mir-64* clusters. However, these miRNAs are unlikely to be misannotated because we identified their homologs in eight *Caenorhabditis* species (supplementary fig. S8, Supplementary Material online and unpublished results).

### PCR Amplification and Sequencing

DNA was amplified for each strain from a single individual using the manufacturer’s protocol of the REPLI-g kit (Qiagen), with the exception of the *C. remanei* strains from Ontario. Genomic DNA isolated from individual worms was diluted 20 times prior to polymerase chain reaction amplification. For each strain from Ontario, DNA was isolated from large populations of worms using the DNeasy Blood and Tissue kit (Qiagen). Primers designed from the reference genome of *C. remanei* strain PB4641 were used to amplify and sequence miRNAs with ∼200 bp of upstream and downstream sequence in *C. remanei* and *C. latens* strains. Amplifications were processed in 30 µl reaction volumes with 1.5 µl dimethyl sulfoxide (DMSO), 3 µl of nucleotide mix (6.6 mM), 3 µl 10X buffer (Fermentas), 2.4 µl MgCl_2_, 0.36 µl of each primer (50 µM), 0.18 µl of Taq polymerase (New England Biolabs), and 2 µl of genomic DNA. Cycling conditions were as follows: 95 °C for 4 min followed by 35 cycles of 95 °C for 1 min, 55 °C or 58 °C for 1 min, and 72 °C for 1 min. Amplifications were sequenced at the University of Arizona sequencing facility. All markers were sequenced on both strands and all polymorphisms were visually verified using sequencing chromatograms. Heterozygous sites were coded according to the International Union of Pure and Applied Chemistry and haplotypes were resolved using the program PHASE 2.1 implemented in DnaSP 5.10 (Librado and Rozas 2009). Primer sequences were manually deleted from each sequence prior to analysis.

### Phylogenetic Conservation and Expression Level of miRNAs

We obtained the distribution of miRNA families from four *Caenorhabditis* species (*C. elegans*, *C. briggsae*, *C. remanei*, and *Caenorhabditis brenneri*) from miRBase v20 and de Wit et al. (2009). We applied a strict criterion to define nonconserved miRNAs in the *C. remanei* genome as those with a unique seed sequence not known from any other *Caenorhabditis*. This procedure yielded 24 “unique” and 105 “conserved” miRNAs in our data set. As an alternative classification, we defined nonconserved miRNAs as those with a seed belonging to a family unique to *C. remanei* or present in at most one other *Caenorhabditis* species, similar to the definition adopted by Shi et al. (2013). By this alternative definition, we classified 29 “low conservation” and 100 “high conservation” miRNAs in our data set. We quantified miRNA expression level as the number of RNA-sequencing reads from de Wit et al. (2009).

### Sequence Analyses

We aligned sequences manually for each locus using BioEdit (Hall 1999) and then quantified SNP using the diversity index π in DnaSP 5.10 (Nei 1987; Librado and Rozas 2009). We also quantified SNP density per base pair as the ratio of the number of SNPs to the length of sequence examined, divided by log(*n*−1), where *n* is the sample size, analogous to Watterson’s correction (Watterson 1975). *Caenorhabditis latens* sequence served as outgroup to determine ancestral and derived alleles within *C. remanei* and to quantify *K*, the interspecies nucleotide divergence between orthologs, with a Jukes–Cantor distance in MEGA 5 (Tamura et al. 2011). For comparison with miRNAs, we compiled published polymorphism data for 78 *C. remanei* protein-coding genes to compute SNP density at synonymous and nonsynonymous sites (Jovelin et al. 2003, 2009; Cutter et al. 2006; Cutter 2008; Jovelin 2009; Dey et al. 2012). A set of 20 coding genes available for multiple populations of *C. remanei* and for *C. latens* was applied to contrasts involving those population samples (Dey et al. 2012). For all analyses of interspecies divergence, we used *C. remanei* strain PB4641 (reference genome) and *C. latens* strain VX0082 (or VX0087 if the VX0082 allele was missing).

We investigated selective constraints in miRNAs by comparing nucleotide polymorphism and divergence in mature miRs, hairpin backbones (hairpin minus mature miR), and synonymous sites of protein-coding genes. We also analyzed polymorphism (π) and divergence (*K*, Jukes–Cantor distance) at the genomic location of miRNAs using overlapping 15-bp long windows with a 5-bp step using DnaSP 5.10. Windows were aligned on the first position of the miRNA sequence and nucleotide diversity and divergence were averaged across each window, eliminating point estimates with <10 windows. For the sliding window analysis, we focused on miRNAs that are >200 bp distant from their nearest miRNA neighbor so that flanking regions do not contain miRNA sequence (*n* = 95 for polymorphism, *n* = 58 for divergence). To examine the structural context of miRNA SNPs, we first predicted, for each miRNA, the stem-loop structure of the PB237 strain allele, or the PB247 allele if the PB237 allele was missing, using the Vienna RNA server (Gruber et al. 2008). We then used the structure of the PB237 allele as a reference to map SNPs in different regions of the hairpin and at paired and unpaired sites. Similarly, we investigated the structural context of substitutions using the hairpin of *C. remanei* strain PB4641 as reference.

We quantified the site frequency spectrum (SFS) in the entire hairpin with Tajima’s *D* (Tajima 1989) and determined its significance by coalescent simulations using DnaSP 5.10 with 50,000 replicates, making the conservative assumption of no intragenic recombination (Tajima 1989; Wall 1999) and assuming a standard-neutral model. The pattern of polymorphism in *C. remanei* suggests demographic equilibrium, particularly in the populations from Ohio and Ontario (Cutter et al. 2006; Dey et al. 2012). We also tested deviation from neutrality using the normalized Fay and Wu’s *H* (Fay and Wu 2000) computed with the program DH and assessed its significance by coalescent simulations with 10,000 replicates (Zeng et al. 2006).

We measured genetic differentiation among populations with *F*_ST_ (Wright 1951) using the entire hairpin sequence. For each SNP in the mature miR, we also quantified the frequency of the derived allele or the frequency of the minor allele using the Ohio population as reference, in three populations of *C. remanei* from Ohio, Ontario, and Germany.

We identified an extra *mir-64* copy within the *C. latens mir-64* cluster based on sequence similarity with *mir-64* homologs in *C. latens* and *C. remanei*, and typical minimum free energy stem-loop structure computed with the Vienna RNA server. We reconstructed the phylogenetic relationships among mature sequences of members of the *mir-64* cluster in *C. remanei*, *C. latens*, *C. elegans*, *C. briggsae*, and *C. brenneri* using Neighbor-Joining with a maximum composite likelihood distance in MEGA 5, and assessed node confidence with 1,000 bootstrap replicates. We also computed nucleotide divergence, with a maximum composite likelihood distance, among *mir-64* homologs in *C. remanei* and *C. latens* separately for the mature and hairpin sequences.

We predicted the hairpin structure of miRNA block563 alleles in *C. remanei* and *C. latens* to examine the effect of a large 51-bp deletion in *C. remanei* on miRNA folding and stability. We extended the analysis of conservation of miRNA sblock26 to *Caenorhabditis japonica*, *C.* sp. 5, and *Caenorhabditis tropicalis* and searched for orthologs in the genome assemblies of these species in Wormbase WS238 using BLAST (Altschul et al. 1990), where *C. tropicalis* was previously known as *C*. sp. 11 (Felix et al. 2014). We identified four conserved sequences in *C. tropicalis* and *C. brenneri*. However, we detected 51 identical hairpin sequences in *C. japonica*, located in contigs ∼2 kb or less in length. We assumed that the large number of identical sequences is an artifact of the current genome assembly and retained only one sequence for further analyses. We estimated pairwise sequence divergence among sblock26 homologs separately for the miR and the entire hairpin with a Jukes–Cantor distance in MEGA 5.

## Results

### Allelic Variation at miRNA Loci in Natural Populations

**Fig. 1.—:**
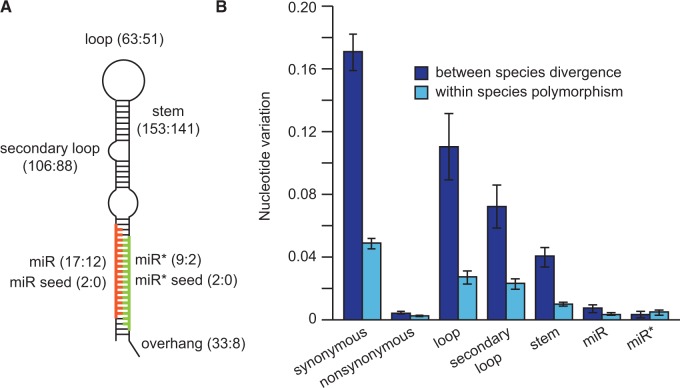
The level of selective constraints varies among different regions of the miRNA hairpin. (*A*) Diagram of a stereotypical miRNA hairpin with a stem-loop structure. The number of mutations segregating in the population from Ohio and the number of substitutions between *Caenorhabditis remanei* and *Caenorhabditis latens* are respectively shown in parentheses for each region of the miRNA (i.e., polymorphisms: substitutions). (*B*) Nucleotide differences in miRNA regions are lower than nucleotide differences at synonymous sites of protein-coding genes. Light blue, within species variation; dark blue, between species divergence; miR, mature miRNA; miR*, star sequence. Means are represented ± 1 standard error of the mean.

We discovered a total of 381 SNPs and 32 indels located within 129 miRNA gene hairpins in an Ohio population of *C. remanei* (fig. 1*A* and supplementary table S1, Supplementary Material online). This gene collection comprises a large majority of the miRNAs in the genome: 83% of miRNAs identified in de Wit et al. (2009) and 69% of miRBase release 20 miRNAs (Kozomara and Griffiths-Jones 2011) that include a recent update of *Caenorhabditis* miRNAs (Shi et al. 2013). Nearly one-third (29%) of miRNA hairpins had no polymorphisms at all, although polymorphic miRNAs contained up to 20 variable sites. Subsequent analysis of *C. remanei* strains from Germany and Ontario revealed respectively 153 and 151 SNPs and 11 and 10 indels in a subset of 38 miRNA genes that we had found to have allelic variation within Ohio (supplementary table S1, Supplementary Material online). And, for the 79 miRNAs that we sequenced in the closely related species *C. latens*, we identified 302 nt substitutions and 37 indel differences between the 2 species. This wealth and density of mutations in miRNA genes within a single species and between sister species thus provide a powerful substrate to test the processes driving their microevolutionary dynamics.

### Patterns of Polymorphism Reveal Selective Constraints on miRNAs

**Fig. 2.—:**
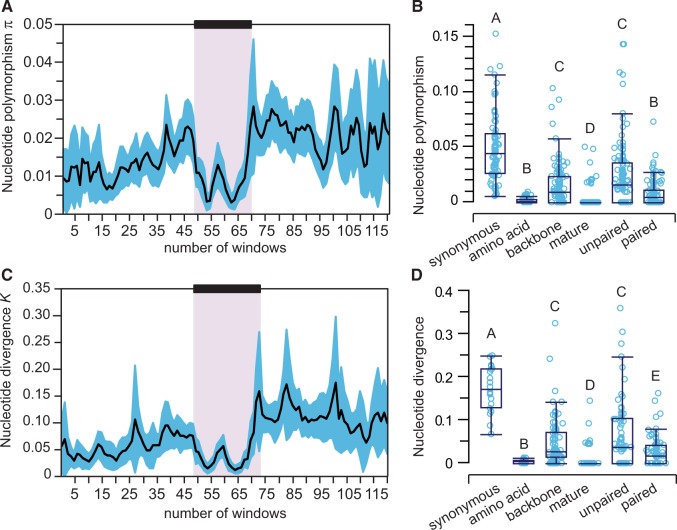
miRNA loci experience strong purifying selection to maintain regulatory interactions and to preserve the integrity of the hairpin structure. Average nucleotide differences within population (*A*) and between species (*B*), calculated using a sliding window, are lower in the region corresponding to the miRNA hairpin, represented by a black box. Black lines indicate mean nucleotide diversity and divergence and the blue areas indicate the 95% confidence interval. Nucleotide differences in the mature miR and in the backbone (hairpin − miR) are lower than nucleotide differences at synonymous sites of protein-coding genes (*C*, *D*). Similarly, both paired and unpaired sites of the miRNA hairpin show signatures of purifying selection when compared with synonymous changes (*C*, *D*). Median polymorphism and divergence are shown by a horizontal line. The box represents the IQR between the first and third quartile. The whiskers extend to the furthest data point within 1.5 times the IQR from the box. Means with different letters are significantly different with Wilcoxon two-sample tests. IQR, interquartile range.

Given this abundance of allelic variants in miRNA sequences, how does the pattern of nucleotide polymorphism implicate natural selection? SNP density differs drastically across structural regions of the miRNAs, indicating strongest selective constraint for the mature and star miRNA regions and lowest constraint for sites located in loops (fig. 1*B*). However, purifying selection reduces nucleotide variation across the entire hairpin sequence, leaving a clear signature relative to genomic locations that flank miRNAs (fig. 2*A* and *C*). When we quantified nucleotide polymorphism and sequence divergence separately for the mature miR in the hairpin, we observed 18 times less polymorphism than for synonymous sites, used as a proxy for selective neutrality. This pattern is indicative of potent purifying selection on miRNA sequences and extends even to miRNA backbone sequences (the hairpin minus the miR), which contain three times less polymorphism than synonymous sites (fig. 2*B* and *D*). The same pattern holds for the populations of *C. remanei* from Ontario and Germany and for nucleotide divergence in orthologous regions between *C. remanei* and *C. latens* (supplementary tables S2–S4, Supplementary Material online).

We further examined patterns of selection on miRNAs using allele frequencies. The majority of SNPs segregate at low frequency when using both the minor and derived allele frequency (MAF and DAF), similar to previous observations in *C. briggsae* (Jovelin and Cutter 2011). The skewed distributions of MAF and DAF further implicate negative selection as the dominant evolutionary force operating on miRNA loci in present-day populations (supplementary fig. S1, Supplementary Material online).

Unpaired sites, comprising the primary hairpin loop and secondary loops that form owing to imperfect base complementarity along the hairpin foldback, have more abundant SNP variation than paired sites (fig. 2*C* and *D*). However, we found that both paired and unpaired miRNA sites show significantly lower nucleotide variation relative to synonymous sites of coding genes (fig. 2*C* and *D*).

For the aggregate analyses described above, we included a set of candidate novel miRNAs identified through RNA-seq (de Wit et al. 2009) that are not currently deposited in miRBase release 20 (see Materials and Methods). We then repeated our analyses separately for miRBase miRNAs and for the candidate miRNAs. We detected qualitatively similar signatures of purifying selection on candidate miRNAs as for miRBase-annotated miRNAs (supplementary fig. S2, Supplementary Material online), providing evidence that the non-miRBase miRNAs may indeed represent functional miRNA genes and that our general conclusions are unaffected by sampling scheme of loci (see also below). Overall, we find that all segments of the miRNA hairpin are subject to selective constraints for the preservation of the hairpin structure and to maintain miRNA-target interactions.

### miRNA Allele Frequency Divergence among Populations

**Fig. 3.—:**
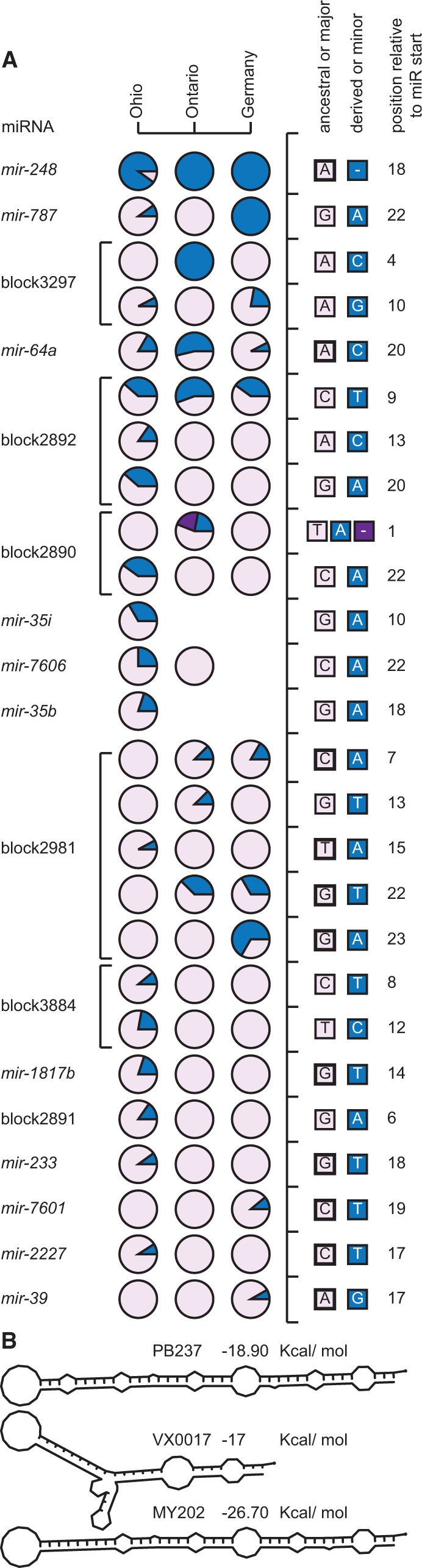
(*A*) SNP frequencies in mature miRs among populations of *C. remanei*. Most variants are found in a single population and at low frequency, with noticeable exceptions for instance for SNPs in *mir-64a*, *mir-248*, *mir-787*, block2892, and block3297. Columns represent separate populations and rows represent distinct SNPs. Each circle represents the frequencies of the ancestral or major allele (in purple) and the derived or minor allele (in blue). The different alleles and their position relative to the start of the mature miR are indicated in the right panels. Ancestral alleles identified by comparison with *C. latens* are marked with a thick line. SNPs located in a same miRNA are joined by a horizontal bar. (*B*) A 14-bp long deletion present in 22% of the population from Ontario removes the seed motif of the mature miR in miRNA block2890 and also alters the hairpin structure.

We contrasted variation in mature miR sequence among *C. remanei* populations to explore the possibility that population-specific variants and differences in allele frequencies between populations could reflect local adaptation to different parts of the species range. Most of the SNPs in mature miRs are unique to a single population and occur at low frequency, indicative of ongoing species-wide purifying selection to eliminate new detrimental mutations. Of the 23 SNPs in miR sequence, however, six appeared in two or all three populations of *C. remanei* (fig. 3*A*). Moreover, eight miRNAs (block2890, block2892, block2981, block3297, *mir-35i*, *mir-64a*, *mir-787*, and *mir-7606*) have SNPs with minor allele frequency ≥25% in at least one population. For instance, the major allele in the Ohio population at position 22 in *mir-787* is fixed in the population sample from Ontario, whereas the minor allele is fixed in the population sample from Germany (fig. 3*A*). Thus, despite the potential contribution to heterogeneity in gene regulation among individuals, most observed SNPs in the mature miRNA may have limited long-term impact on the evolution of gene regulation. And yet, our analysis also identifies candidate miRNAs that may be involved in differential gene expression among populations.

**Fig. 4.—:**
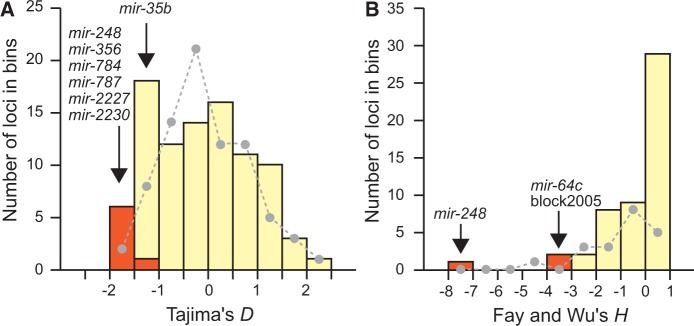
Nonneutral pattern of sequence variation in miRNA hairpins. miRNAs with SFS deviating from neutral expectations with Tajima’s *D* (*A*) and Fay and Wu’s *H* (*B*) are labeled in orange, and miRNAs with SFS compatible with neutrality are labeled in ivory. The distributions of *D* and *H* for protein-coding genes are shown for comparison with dashed lines.

We evaluated allele frequencies within the Ohio population quantitatively using Tajima’s *D* (*D*_Taj_) and Fay and Wu’s *H* (*H*_FW_) statistics to assess skews in the SFS that can be indicative of natural selection on miRNA sequences (Tajima 1989; Fay and Wu 2000). We detected nonneutral patterns of nucleotide variation in nine genes, four of which have an SNP located in the mature miR (*mir-35b*, *mir-248*, *mir-787*, and *mir-2227*). SNPs in seven miRNAs show an excess of rare variants (*D*_Taj_ < 0 for *mir-35b*, *mir-248*, *mir-356*, *mir-784*, *mir-787*, *mir-2227*, and *mir-2230*; fig. 4*A*) and three miRNAs have an excess of derived high-frequency alleles (*H*_FW_ < 0 for *mir-64c*, *mir-248*, and block2005; fig. 4*B*). These tests are significant only for *mir-248* and *mir-35b* after applying the Benjamini–Hochberg correction for multiple testing with a 5% false discovery rate. Note that the miRNAs with nonneutral patterns of diversity identified with the *D*_Taj_ and the *H*_FW_ statistics are largely nonoverlapping because of the lack of an available orthologous outgroup for most of the miRNAs with significant skewed SFS. Nevertheless, this discrepancy could also reflect differences between the two tests because negative *D*_Taj_ values are compatible with the action of negative and positive selection, whereas negative *H*_FW_ values typically indicate positive selection. Six of these eight miRNAs with nonneutral patterns of variation have homologs in *C. elegans*. To gain insights into the potential function of these six miRNAs, we obtained the lists of all predicted target genes in *C. elegans* using TargetScanWorm 6.2 (Jan et al. 2011), and determined functional enrichment of Gene Ontology (GO) terms related to biological processes using DAVID (Huang et al. 2009). Interestingly, the first ranked functional clusters of five miRNAs (*mir-64*, *mir-248*, *mir-356*, *mir-784*, and *mir-787*) are enriched for GO terms related to gonad development, although enrichment is significant only for *mir-248*, *mir-356*, and *mir-787* after correction for multiple testing (supplementary table S5, Supplementary Material online). The SFS for SNPs in flanking regions of *mir-248*, *mir-784*, and *mir-2230* also deviate from neutrality, so we could not precisely identify the genomic loci with perturbed SFS. Nevertheless, nongenic regions of these miRNAs (supplementary fig. S3, Supplementary Material online) could contain *cis*-regulatory sites affecting miRNA transcription, which also may represent important targets of selection.

### Mutations of miR Seed Regions

Despite generally conservative evolution of miRNA genes, the mature miRs contain a greater density of SNPs than do amino acid replacement sites in protein-coding genes (supplementary table S2, Supplementary Material online). Four mature miRs (block2891, block2981, block3297, and block3884) contain an SNP in the seed motif required for target interaction (fig. 3*A*), subject to some caveats discussed below. However, these SNPs are present at moderate frequency suggesting that they may have limited impact on the evolution of miRNA function. One exception is the derived A → C transversion in the seed of miRNA block3297, which occurs in all three strains from Ontario but is absent in the samples from Ohio and Germany (fig. 3*A*). Nevertheless, the small sample size for this miRNA in the population from Ontario makes it unclear whether this SNP is truly fixed or if this site might prove variable if other individuals could be assessed. Two other miRNAs (*lin-4* and *mir-64b*) have an SNP in the seed motif of the star miRNA, although we observed no substitutions between *C. remanei* and *C. latens* orthologs in the seeds of mature and star miRNAs (fig. 1*A*).

Structural variants involving miRNAs also can generate substantial functional diversity. We found indel polymorphisms that completely delete the seed sequence or mature miR for three other miRNAs, providing examples of variation in miRNA content within and between populations of a single species. A 14-bp deletion, unique to the Ontario sample, removes the entire seed and strongly destabilizes the miRNA hairpin (fig. 3*B*). Another indel is a 59-bp long deletion removing the entire mature miR, present in 20% of the strains from the Ohio sample (supplementary fig. S4, Supplementary Material online). The other deletion is 201-bp long and removes 89% of the hairpin sequence in all strains from Ohio (supplementary fig. S5, Supplementary Material online).

### Evolution of Novel and Lineage-Restricted miRNAs

**Fig. 5.—:**
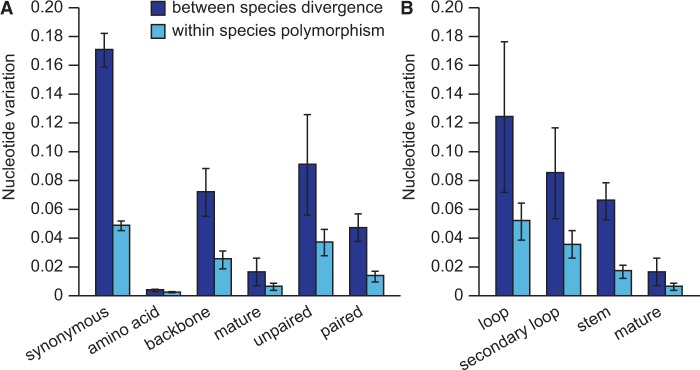
Signatures of purifying selection in miRNAs belonging to novel families unique to *C. remanei* and *C. latens*. (*A*) Selective constraints are stronger for mature sequences and paired sites. (*B*) Distribution of nucleotide variation in different region of miRNA hairpins.

We next sought to address the relationship between phylogenetic conservation and sequence polymorphism. When we classify *C. remanei* miRNAs based on the degree of preservation of their family in other *Caenorhabditis* species (unique vs. conserved), we find that evolutionary signatures both in the short term (polymorphism) and longer term (divergence) are concordant with faster evolution of those miRNAs unique to *C. remanei* and *C*. *latens* (supplementary fig. S6, Supplementary Material online). However, we detect qualitatively similar signatures of purifying selection both for unique and conserved miRNAs (fig. 5). Altogether, these findings are consistent with more rapid sequence evolution for newly emerging miRNAs, as observed in *Drosophila* (Nozawa et al. 2010; Mohammed et al. 2013; Lyu et al. 2014), nevertheless coupled to selection favoring their persistence.

**Fig. 6.—:**
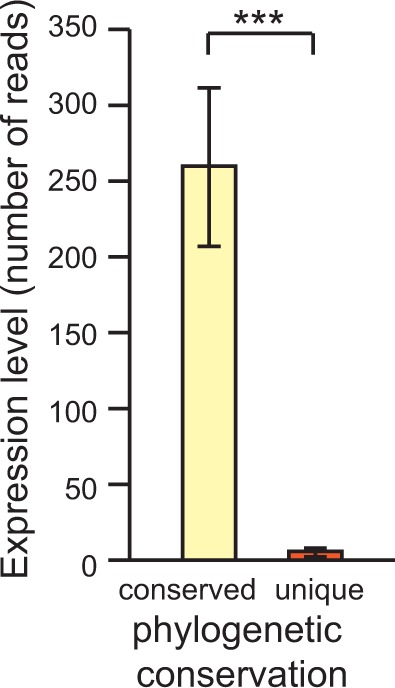
miRNAs from families unique to *C. remanei* are expressed at lower levels than miRNAs from families conserved in other *Caenorhabditis* species. Expression read counts from de Wit et al. (2009).

The evolution of novel miRNAs could have genome-wide consequences on gene regulation owing to the small motifs of sequence complementarity required for binding to potential targets (Bartel 2009; Zheng et al. 2011). However, low and/or restricted spatio-temporal expression of newly emerging miRNAs could limit the deleterious effects of novel regulatory interactions (Chen and Rajewsky 2007). Consistent with this hypothesis, we found that phylogenetically restricted miRNAs have significantly lower expression in *C. remanei* (fig. 6). In addition, miRNA expression level in *C. remanei* correlates negatively with the amount of nucleotide variability (hairpin: Spearman’s *ρ* = −0.214, *P* = 0.017; backbone: *ρ* = −0.207, *P* = 0.021; miR: *ρ* −0.221, *P* = 0.014). A qualitatively similar, nonsignificant trend involving miRNA expression level is present for sequence divergence between orthologs in *C. remanei* and *C. latens* (hairpin: *ρ* = −0.184, *P* = 0.110; backbone: *ρ* = −0.186, *P* = 0.106; miR: *ρ* = −0.206, *P* = 0.072). These results suggest that low expression of novel miRNA genes could delay their elimination and may enable their integration into regulatory networks while also allowing faster sequence evolution, perhaps facilitated by positive selection (Lyu et al. 2014).

In vertebrates, new miRNAs tend to arise in intronic sequences (Campo-Paysaa et al. 2011; Meunier et al. 2013), and so miRNA genes nested in the introns of other genes might be younger and show faster sequence evolution than miRNAs located in intergenic regions. However, we find that miRNAs unique to the *C. remanei* lineage are no more abundant in introns than are miRNAs conserved across many species (χ^2^ *P* = 0.76), with approximately one-fifth of both classes occurring in introns. Moreover, SNP variation and divergence do not differ significantly between miRNAs occurring in introns versus intergenic locations, or, it is the intergenic miRNAs that show more sequence differences (supplementary fig. S7, Supplementary Material online). This result is qualitatively consistent with comparisons of substitution rates between orthologous miRNAs in *C. briggsae* and *C. nigoni* (formerly *C*. sp. 9) (Jovelin 2013).

### Evaluation of Possible miRNA Misannotation

The analyses presented above identify several miRNAs with alleles that are predicted to affect the regulation of their target genes despite overall strong purifying selection on miRNA loci, and suggest different patterns of selection for young and phylogenetically conserved miRNAs. Because these analyses single out specific miRNAs, we tested the robustness of our results against possible miRNA misannotation by removing 12 miRBase and 9 non-miRBase miRNAs that exhibited greater nucleotide diversity than in sites directly flanking the hairpin. An incidental byproduct of this procedure is that our SNP data provide evidence of selective constraint for 11 non-miRBase loci, indicative of them representing legitimate miRNA genes. This procedure eliminates two miRNAs with SNPs in the seed region (block2981 and block3297) and two miRNAs with skewed SFS (*mir-35b* and block2005). However, the pruned data set still contains miRNAs with population-specific alleles and miRNAs with signatures of adaptive evolution, although only one miRNA shows a significant excess of derived high-frequency SNPs after correction for multiple testing (supplementary fig. S8*A*, *F*, and *G*, Supplementary Material online). Moreover, our conclusions that young miRNAs evolve faster and are expressed at lower level than phylogenetically conserved miRNAs while showing signatures of negative selection also remain robust to the potential of misannotation (supplementary fig. S8*B–E*, Supplementary Material online). Thus, our general conclusions about miRNA microevolution are robust to how miRNA loci are sampled in the genome with respect to potential gene misannotations.

### Rapid Evolution of a miRNA Gene Cluster

**Fig. 7.—:**
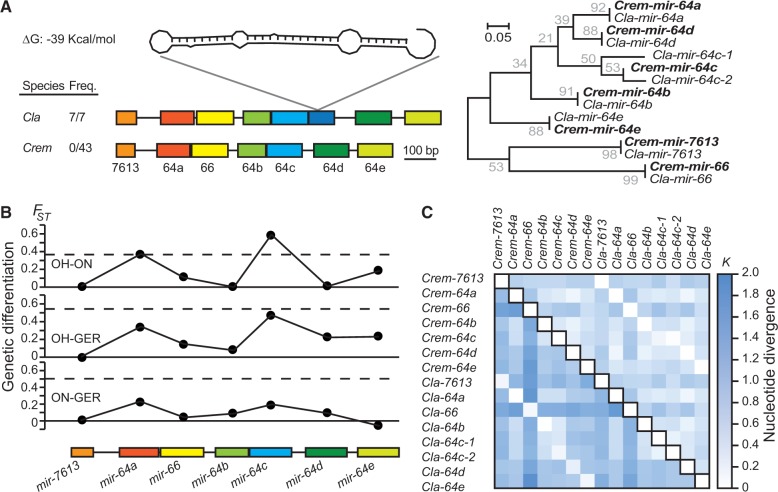
Rapid evolution of a miRNA cluster. (*A*) The *mir-64* cluster expanded by ancient and recent tandem duplications (supplementary fig. S8, Supplementary Material online). The number of *mir-64* paralogs differs between *C. remanei* and *C. latens* because of the possible loss of *mir-64c-1* in *C. remanei*. (*B*) Genetic differentiation of *mir-64* members among three populations of *C. remanei*. *mir-64c* is highly differentiated between samples from Ohio and Ontario, suggesting that *mir-64c* may be the target of adaptive evolution. The dash line represents the 95 percentile of *F*_ST_ values in a set of protein-coding genes. (*C*) Distance matrices between paralogs and orthologs in *C. remanei* and *C. latens* for the entire hairpin sequence (below diagonal) and for the mature sequence (above diagonal). *Crem*, *C. remanei*; *Cla*, *C. latens*.

miRNAs are often organized along the genome in clusters that may be transcribed as a polycistronic unit (Baskerville and Bartel 2005; Axtell et al. 2011). Clustered miRNAs can experience rapid evolution in terms of species-specific tandem duplications, losses, and single nucleotide substitutions in the seed motif of homologs, as seen among species in *Drosophila* (Mohammed et al. 2013) and in *Caenorhabditis* (de Wit et al. 2009; Jovelin and Cutter 2011; Jovelin 2013; Shi et al. 2013). We found that evolution of the *mir-64* cluster is particularly dynamic. Seven miRNA genes comprise this cluster in *C. remanei*, but *C. latens* contains an eighth miRNA owing to recent tandem duplication of *mir-64c* (fig. 7*A*). Phylogenetic analysis suggests that the duplication of *mir-64c* predates speciation between *C. remanei* and *C. latens* and that *mir-64c-1* was subsequently lost in *C. remanei* (fig. 7*A*), although it remains possible that *mir-64c* duplicated more recently in the lineage leading to *C. latens* (fig. 7*A* and supplementary fig. S9, Supplementary Material online). We detected *mir-64c-1* in all 7 strains of *C. latens* isolated from China and in none of the 43 strains of *C. remanei* from North America, Europe, and Japan, implicating the presence versus absence of *mir-64c-1* as a fixed difference between these species.

Extensive sequence differences in the hairpin and in the mature sequences of *mir-64* paralogs suggest possible subfunctionalization following their origin by duplication (fig. 7*B*), although conservation of the seed in all members implies an overlapping set of target genes. Mature sequences of *mir-64* homologs are well conserved between *C. remanei* and *C. latens*, with the exception of *mir-64c* homologs, which differ by up to three substitutions between the two species. To better understand the very recent evolution of the *mir-64* cluster, we then examined the pattern of genetic differentiation of *mir-64* miRNAs between populations of *C. remanei*. We observed stronger differentiation between the Ohio and Ontario populations for two miRNAs in the cluster than is seen for 95% of loci in a reference set of coding genes (*mir-64a* and *mir-64c*; fig. 7) (Dey et al. 2012). In addition, *mir-64c* is one of the most genetically differentiated miRNAs among the 38 miRNA genes that we analyzed for all 3 populations of *C. remanei*, further implicating this locus in ongoing selectively driven change (supplementary fig. S10, Supplementary Material online).

## Discussion

We have conducted the first global survey of miRNA microevolution in *Caenorhabditis*, quantifying allelic nucleotide variability for the majority of the miRNA genes in the *C. remanei* genome. Investigations of miRNA evolution in *Caenorhabditis* have so far been limited to changes in small RNA composition between species, interspecies divergence in sequence, or to polymorphism in a small subset of miRNAs (de Wit et al. 2009; Jovelin and Cutter 2011; Jovelin 2013; Shi et al. 2013). Even beyond nematodes, astoundingly few studies have attempted to characterize evolutionary pressures on contemporary timescales for miRNAs (Saunders et al. 2007; Ehrenreich and Purugganan 2008; Liang and Li 2009; Quach et al. 2009). We discovered 413 natural allelic variants from a single population located in the hairpins of these 129 miRNAs, which contrasts with just 65 alleles described in an analysis of 474 human miRNA hairpins (Saunders et al. 2007; Quach et al. 2009). Our analysis of this rich SNP variation in *C. remanei* demonstrates that contemporary selection pressure on all parts of miRNAs are dominated by purifying selection to eliminate new mutations, even on loop regions of the pre-miRNA hairpin that evolve more rapidly than the mature miR and miR* portions of miRNA genes. However, we also document a surprising abundance of miRNA alleles in nature that are predicted to alter miRNA regulation or function with consequent modulations of regulatory networks involving miRNA targets. This allelic variation includes SNPs in mature miRs, SNPs in seed motifs and paired hairpin sites, as well as indels that ablate some or all of a functional miRNA domain, with an unexpected signature of positive directional selection favoring alternative alleles for nine miRNA genes. Moreover, we characterize rapid change in gene content for the *mir-64* miRNA cluster since the common ancestor of *C. remanei* and its sister species *C*. *latens*, and provide population genetic evidence that contemporary selection acts generally to retain lineage-specific “novel” miRNAs in spite of their more rapid evolution. These findings support the idea that changes in gene expression, mediated through divergence in miRNA regulation over genetic networks, can contribute to phenotypic novelty and adaptation to species-specific selection pressures.

### Selection across miRNA Functional Domains

It is well known from deep-time phylogenetic comparisons that strong purifying selection acts on mature miRNAs (Fahlgren et al. 2010; Mohammed et al. 2013), but it remains less clear whether this mode of selection also predominates in modern day populations. We demonstrate that selection on short and long timescales, in fact, is largely consistent: Strong purifying selection acts on mature miRNA sequence in contemporary populations, not just being evident historically from phylogenetic comparisons. Moreover, we show how all sequence domains within *C. remanei* miRNA genes experience purifying selection that results in conservative evolution, including the somewhat faster evolving unpaired sites in the miRNA hairpin (e.g., loop nucleotides). The distribution of nucleotide changes across the hairpin is broadly consistent with deeper time analyses in other animals and plants (Saunders et al. 2007; Ehrenreich and Purugganan 2008; Quach et al. 2009; Jovelin and Cutter 2011; Mohammed et al. 2013). Sequence polymorphism within species and divergence between species in all these domains is less than half that seen for synonymous sites of coding genes, a putatively neutral reference, with the mature miR and miR* sequences having respectively 18-fold and 11-fold lower SNP density than synonymous sites. This suggests that the mature miR is not the sole bioactive RNA species generated by processing of the miRNA hairpin, but provides an evolutionary signature consistent with recent findings from functional studies that the complementary miR* and loop nucleotides can also have regulatory activity with their own sets of gene targets (Okamura et al. 2008, 2013; Yang et al. 2011; Winter et al. 2013). Moreover, loop regions might also influence gene regulation by controlling miRNA maturation and/or by enabling miRNA hairpins to direct repression (Liu, Min, et al. 2008; Trujillo et al. 2010; Yue et al. 2011; Chen 2013). Our findings therefore implicate selection acting both to maintain the integrity of the hairpin structure for proper biogenesis and to maintain miRNA sequence for proper target specificity.

### miRNA Genes as Candidate Targets of Adaptive Evolution

Deep-time preservation and sequence constraint dominate the prevailing view of miRNA evolution. Despite strong purifying selection to preserve the sequence of most miRNA genes, surprisingly we identified nine miRNAs with unusual signatures of allele frequencies that may be indicative of adaptive evolution (two miRNAs after correction for multiple testing). Our results mirror a study of human miRNAs that identified several instances of positive selection under a global background of purifying selection on miRNA genes (Quach et al. 2009). To learn more about these nine miRNAs, we interrogated the function of their homolog in *C. elegans*. Three of the six miRNAs conserved between *C. remanei* and *C. elegans* (*mir-248*, *mir-784*, and *mir-787*) are located on the X chromosome and five of them (*mir-64*, *mir-248*, *mir-356*, *mir-784*, and *mir-787*) regulate target genes enriched in gonad formation in *C. elegans*. To the extent that the function of these four miRNAs is conserved between *C. elegans* and *C. remanei*, these results are consistent with rapid sequence evolution for X-linked miRNAs and/or miRNAs with sexually selected roles in testis development (Zhang et al. 2007; Guo et al. 2009; Jovelin 2013; Lyu et al. 2014).

In addition, our analysis of the *mir-64* cluster of miRNA genes further implicates *mir-64c* as a direct target of positive selection. We showed that an excess of derived high-frequency variants localized to *mir-64c*, and that *mir-64c* homologs are the only members of this cluster to have substitutions in the mature miR between *C. remanei* and *C. latens*. Moreover, *mir-64c* is the most highly differentiated miRNA in this cluster among populations of *C. remanei*, also being one of the most highly differentiated miRNAs in our data set, and even more strongly differentiated among populations than 95% of non-miRNA reference genes in the genome. The *mir-64c* miRNA is part of a conserved cluster that arose by tandem duplication before the last common ancestor of *C. remanei* and *C. elegans*. However, the number of *mir-64* paralogs varies up to 2-fold because of the independent gain and/or loss of tandem duplicates in different *Caenorhabditis* species. Same-family miRNA members regulate largely overlapping target genes, acting in part redundantly in genetic pathways. Additionally, clustered miRNAs tend to regulate proteins that directly interact and that are part of the same network module (Yuan et al. 2009; Wang et al. 2011), but may also regulate distinct components of genetic pathways (Friggi-Grelin et al. 2008). Tandem duplicates and gene loss within the *mir-64* cluster may modulate miRNA dosage to strengthen gene expression regulation according to species-specific needs (Doench and Sharp 2004). Nevertheless, we also showed extensive sequence divergence in the hairpin and mature sequences of *mir-64* paralogs in both *C. remanei* and *C. latens*. Duplication may enable the fixation of mutations that confer differences in target repression among paralogs, a form of genetic subfunctionalization (Lynch and Force 2000). For instance, functional diversification of human paralogs *mir-181a* and *mir-181c* is largely driven by differences in loop nucleotides (Liu, Min, et al. 2008).

### Evolution of Lineage-Specific miRNAs

Sequencing of small RNAs in nematodes revealed that each species possesses a unique repertoire of miRNA families in addition to conserved miRNAs (de Wit et al. 2009; Shi et al. 2013), a feature also common to primates (Berezikov et al. 2006), fruit flies (Lu, Shen, et al. 2008), and plants (Fahlgren et al. 2010). These species-specific pools of miRNAs imply recent acquisition of new miRNA genes and, consequently, high rates of gene birth and death. We observed this process in the *mir-64* cluster as an ongoing evolutionary dynamic between the closely related species *C. remanei* and *C*. *latens*, and within and between populations of *C. remanei* for three other miRNAs. In humans, several hundred miRNAs are located in copy number variation (CNV) regions. Most miRNAs in high-confidence CNV regions arose in primates and a few are conserved among vertebrates (Marcinkowska et al. 2011). Similarly, we found that only one of the three miRNAs with deletion polymorphism in *C. remanei* is conserved in multiple *Caenorhabditis* species.

We observed selective constraint among evolutionarily young miRNAs, but also that they are expressed at lower level and accumulate more nucleotide polymorphisms than phylogenetically widespread miRNAs. These findings are consistent with the transcriptional control hypothesis that proposes low expression level and/or breadth at birth of novel miRNAs will limit their potential for deleterious misregulation (Chen and Rajewsky 2007; Roux et al. 2012; Meunier et al. 2013; Barbash et al. 2014). Moreover, our results suggest that novel miRNAs may be implicated in regulatory differences among *Caenorhabditis* species, and highlight the potential role of new miRNA genes in evolution. However, young miRNAs in *C. remanei* are not over-represented in introns, in contrast to vertebrate genomes (Campo-Paysaa et al. 2011; Meunier et al. 2013), and intronic miRNAs do not accumulate more mutations than intergenic miRNAs.

## Conclusion

In this study, we have systematically quantified allelic variation to characterize distinct modes of selection for the majority of known miRNAs in the nematode *C. remanei*. Our findings support the idea that changes in gene regulation mediated through divergence in miRNA sequence and miRNA content can contribute to phenotypic novelty and adaptation to specific environments in contemporary populations, in addition to deep phylogenetic bursts of evolution. Because miRNAs can regulate hundreds of genes, mutations in target gene binding sites ought to confer less pleiotropic effects than mutations to the *trans*-acting miRNA. To date, the limited analyses of miRNA binding site polymorphism suggest that many SNPs could cause variation in gene expression (Kim and Bartel 2009), despite pervasive purifying selection (Chen and Rajewsky 2006; Saunders et al. 2007; Ehrenreich and Purugganan 2008), which could then be the molecular focus of adaptive evolution (Loh et al. 2011; Li et al. 2012). Future investigations that integrate variation in miRNAs with their target binding sites to assess coevolutionary dynamics will prove important in further dissecting miRNA regulation in developmental evolution (Tang et al. 2010; Barbash et al. 2014).

## Supplementary Material

Supplementary figures S1–S10 and tables S1–S5 are available at *Genome Biology and Evolution* online (http://www.gbe.oxfordjournals.org/).

Supplementary Data
