# Supplementary material for: Microevolution of Nematode miRNAs Reveals Diverse Modes of Selection
Source: Genome Biol Evol. 2014 Oct 28;6(11):3049–63. doi: 10.1093/gbe/evu239 (PMC4255771; doi:10.1093/gbe/evu239)
Supplement: Supplementary Data [file supp_6_11_3049__index.html]

Microevolution of nematode miRNAs reveals diverse modes of selection — Microevolution of Nematode miRNAs Reveals Diverse Modes of Selection — Supplementary Data 

# Microevolution of Nematode miRNAs Reveals Diverse Modes of Selection

## Supplementary Data

files

**Files in this Data Supplement:**

- Supplementary Data - pdf file
