# Supplementary material for: Microevolution of Nematode miRNAs Reveals Diverse Modes of Selection
Source: Genome Biol Evol. 2014 Oct 28;6(11):3049–63. doi: 10.1093/gbe/evu239 (PMC4255771; doi:10.1093/gbe/evu239)
Supplement: Supplementary Data [file supp_evu239_RJ_mirvar_SuppMat_100214.pdf]

Supplementary Material for:

Richard Jovelin and Asher D. Cutter

Microevolution of nematode miRNAs reveals diverse modes of selection

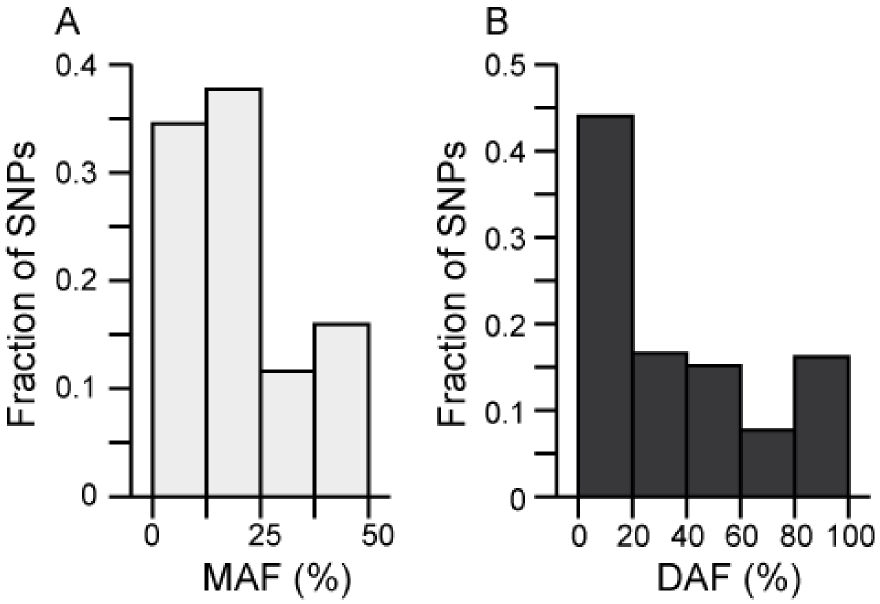

Supplementary Figure 1. The skewed distributions of minor allele frequencies (MAF; A) and derived allele frequencies (DAF; B) are consistent with the action of purifying selection on miRNAs.

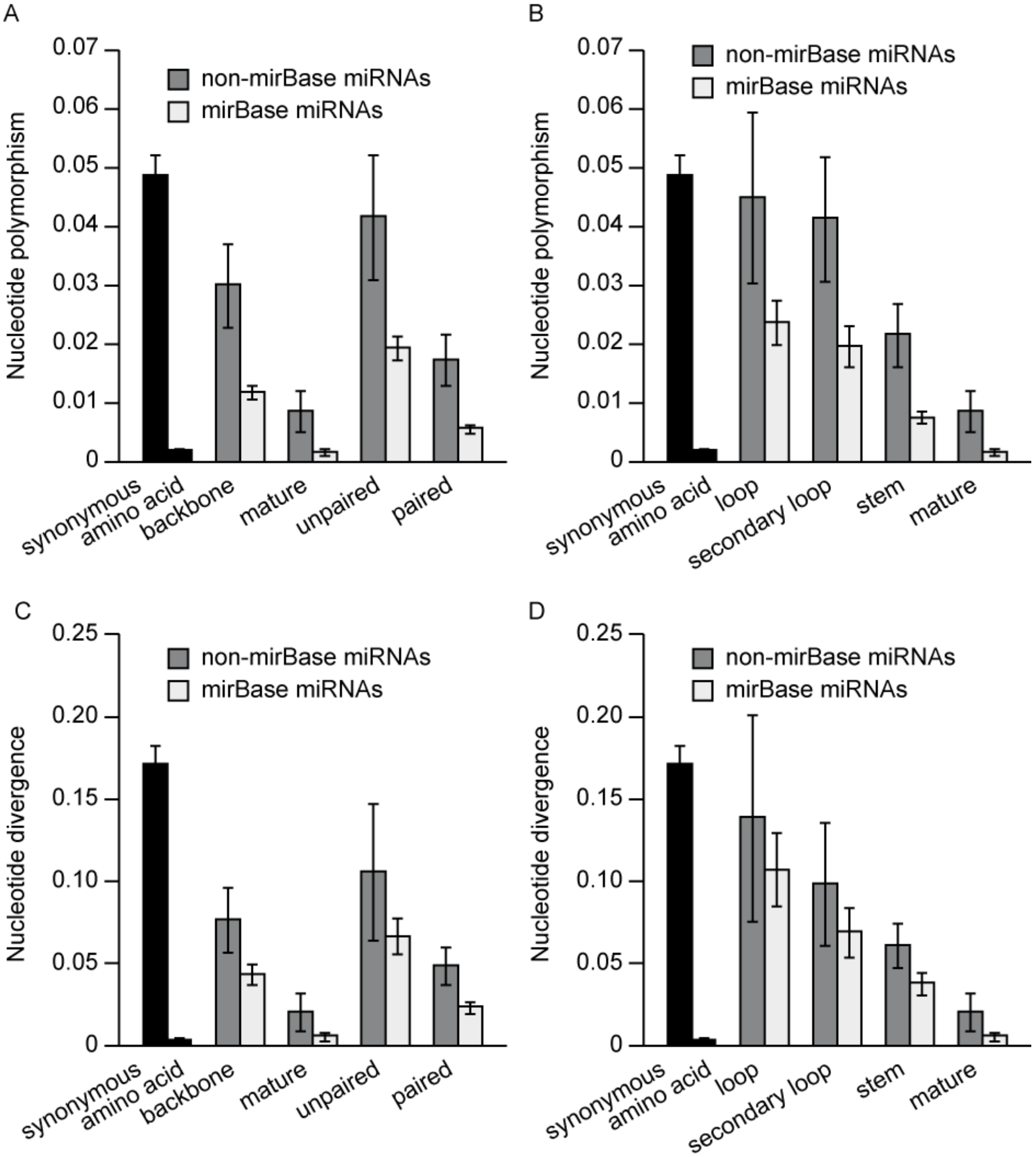

Supplementary Figure 2. Non-mirBase miRNAs show signatures of purifying selection. Although non-miRBase miRNAs are more diverse than mirBase miRNAs, they show similar patterns of selective constraints on different portions of the miRNA hairpins for polymorphism within *C. remanei* (A, B) and divergence between *C. remanei* and *C. latens* (C, D).

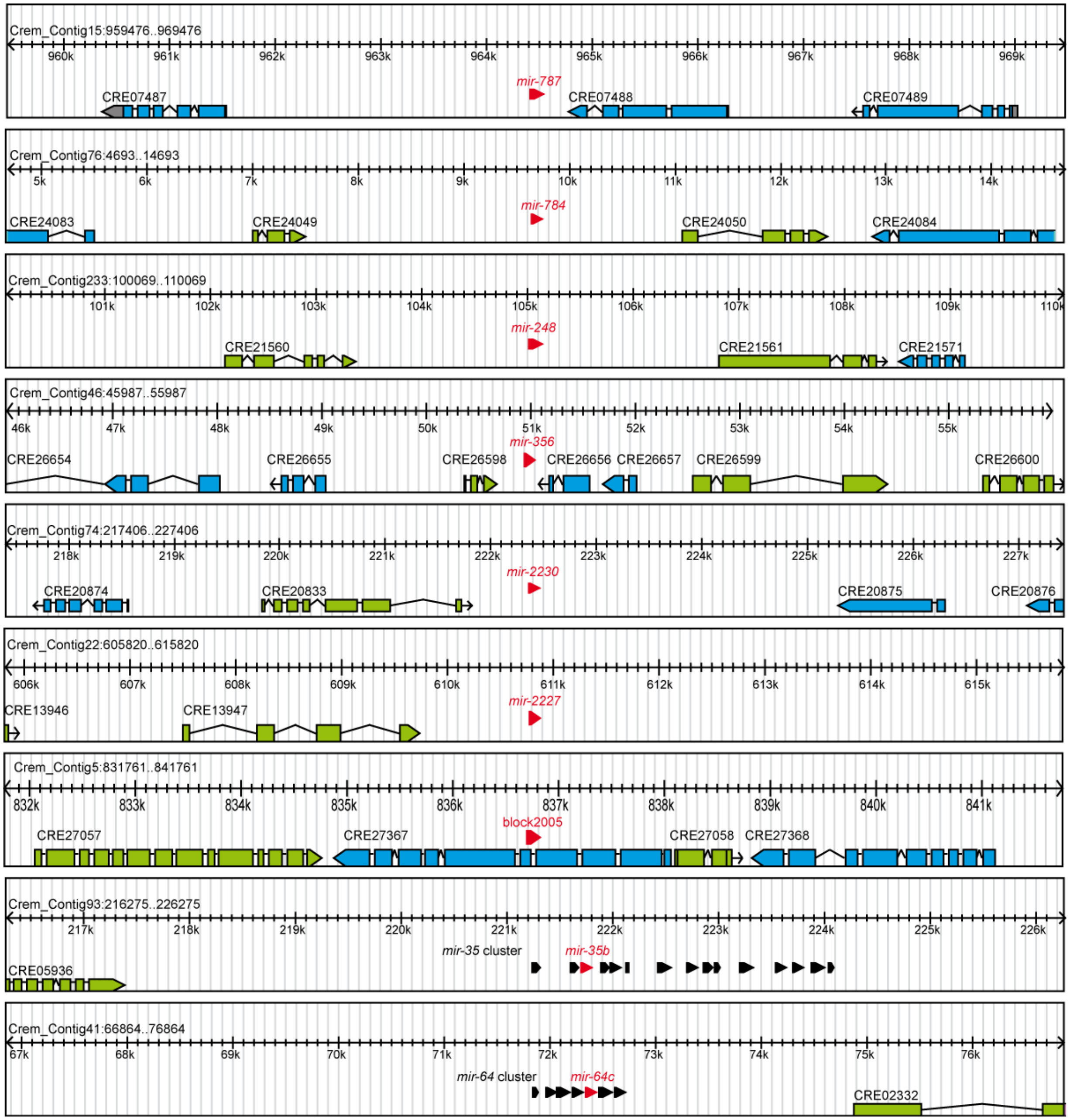

Supplementary Figure 3. Genomic context of miRNAs with signatures of positive selection (in red).

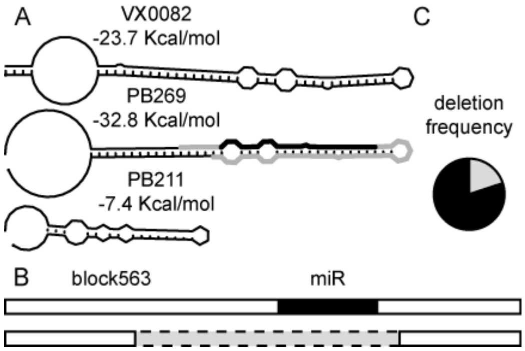

Supplementary Figure 4. miRNA deletion polymorphism within population. A 59 bp-long deletion (grey) removes the entire mature sequence (black), the loop nucleotides and the miR* in miRNA block563, resulting in a short and unstable hairpin (A and B). The deletion is present in 2 of 10 strains sampled (20%) in the population of *C. remanei* from Ohio.

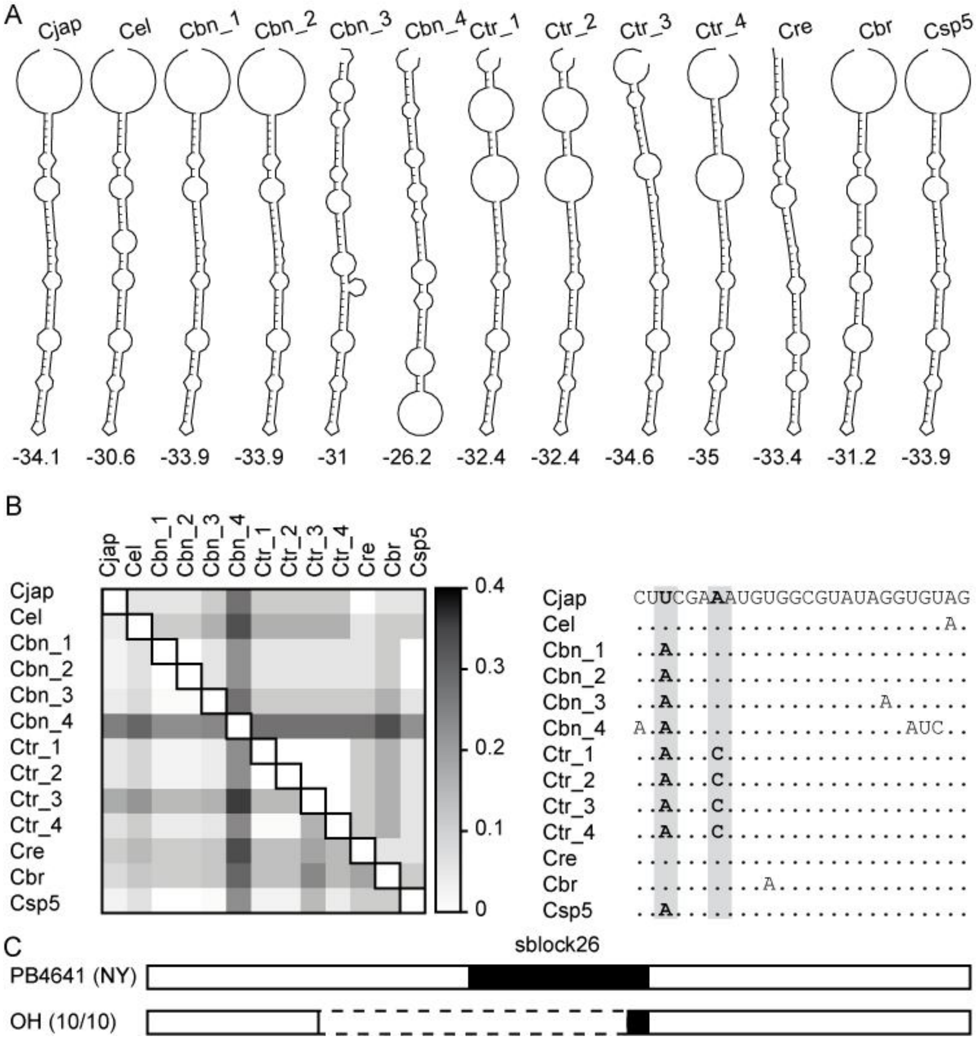

Supplementary Figure 5. miRNA deletion polymorphism among populations of *C. remanei*. A. miRNA sblock26 is conserved across *Caenorhabditis* species. B. The hairpin sequences (below

diagonal) and the mature sequences (above diagonal) of homologs are overall well conserved, but substitutions in the seed motif predict functional divergence. C. A 201 bp-long deletion removing 89 % of the hairpin sequence, including the miR, is present is all strains sampled from the population of *C. remanei* from Ohio. *Cjap: C. japonica; Cel: C. elegans; Cbn: C. brenneri; Ctr: C. tropicalis; Cre: C. remanei; Cbr: C. briggsae*

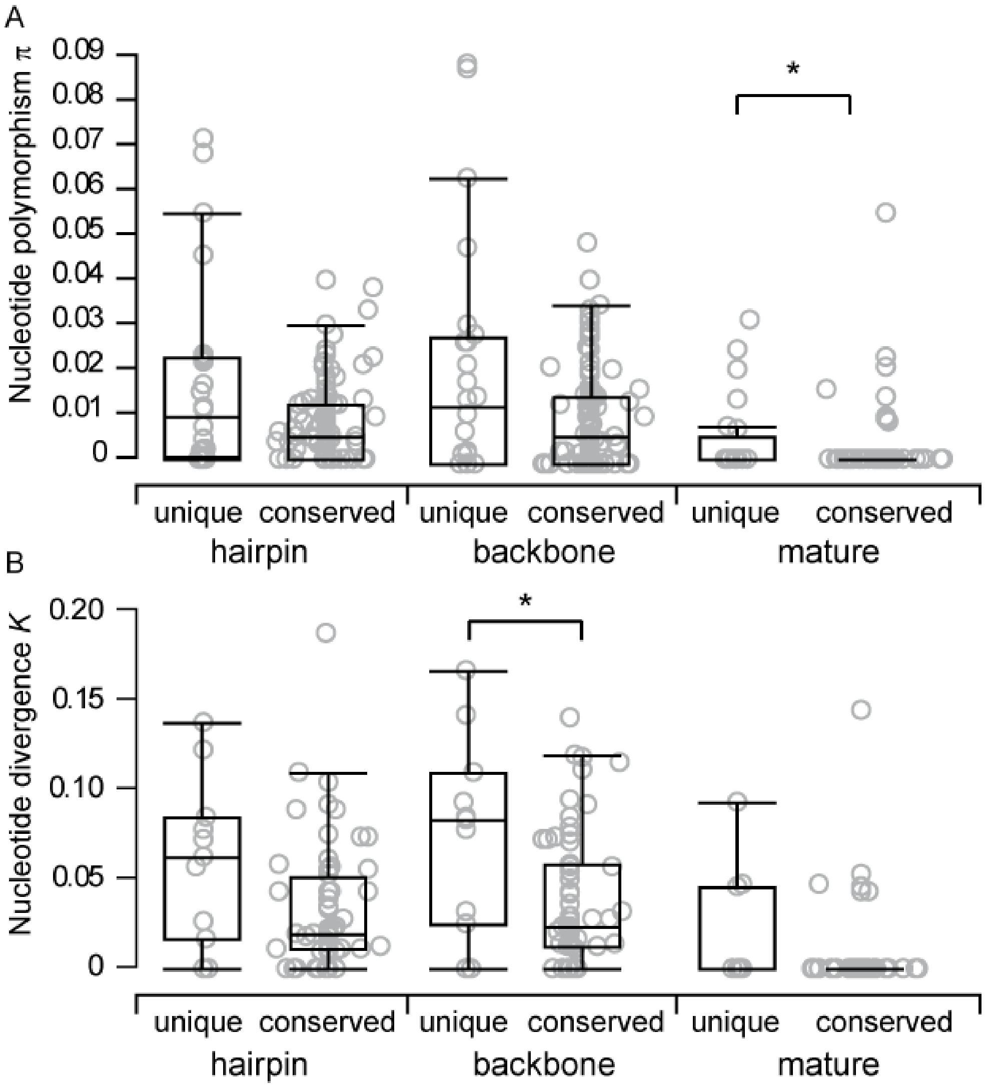

Supplementary Figure 6. miRNAs from families unique to *C. remanei* and *C. latens* have more polymorphisms (A) and divergence (B) than miRNAs from families conserved in other *Caenorhabditis* species.

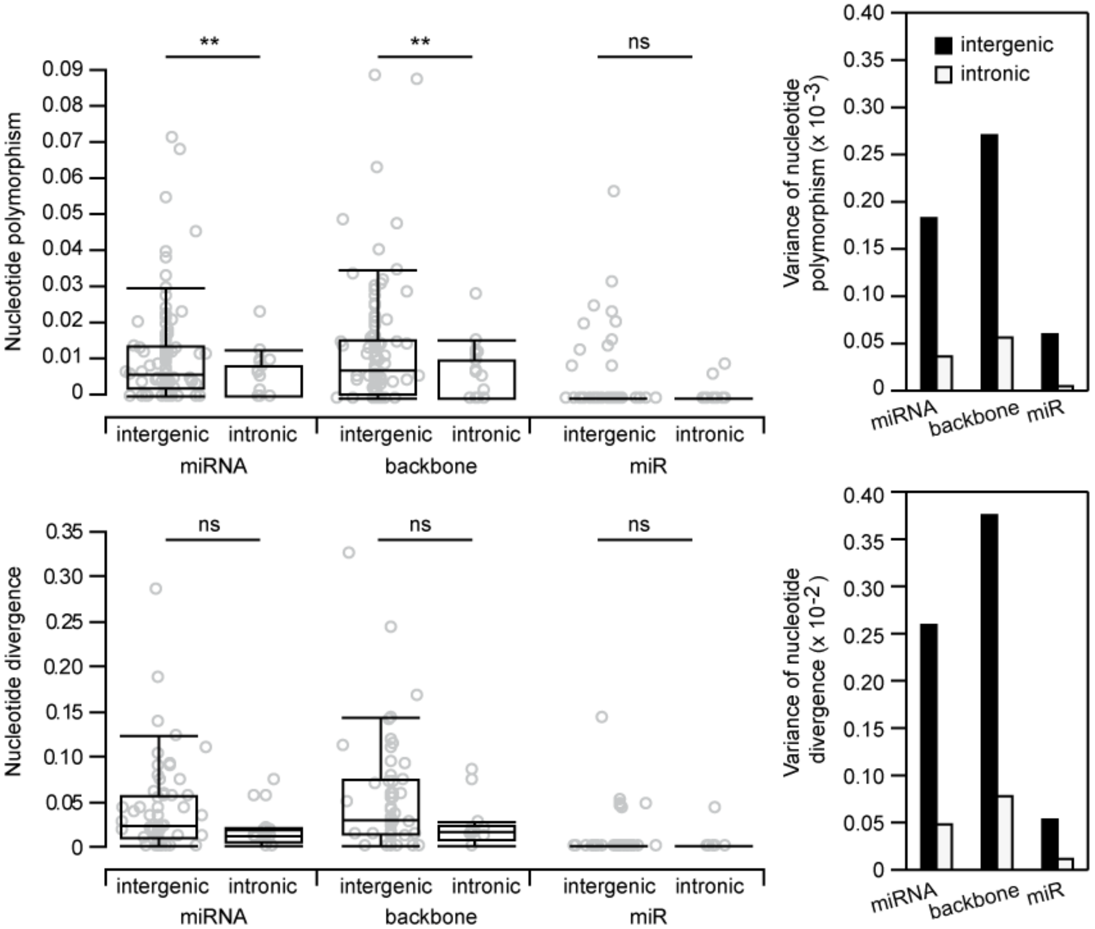

Supplementary Figure 7. Intronic miRNAs are more conserved and have lower variance in nucleotide diversity than intergenic miRNAs. Top: polymorphism within the Ohio population of *C. remanei*. Bottom: Divergence between *C. remanei* and *C. latens*. ** $P < 0.01$. ns: non-significant

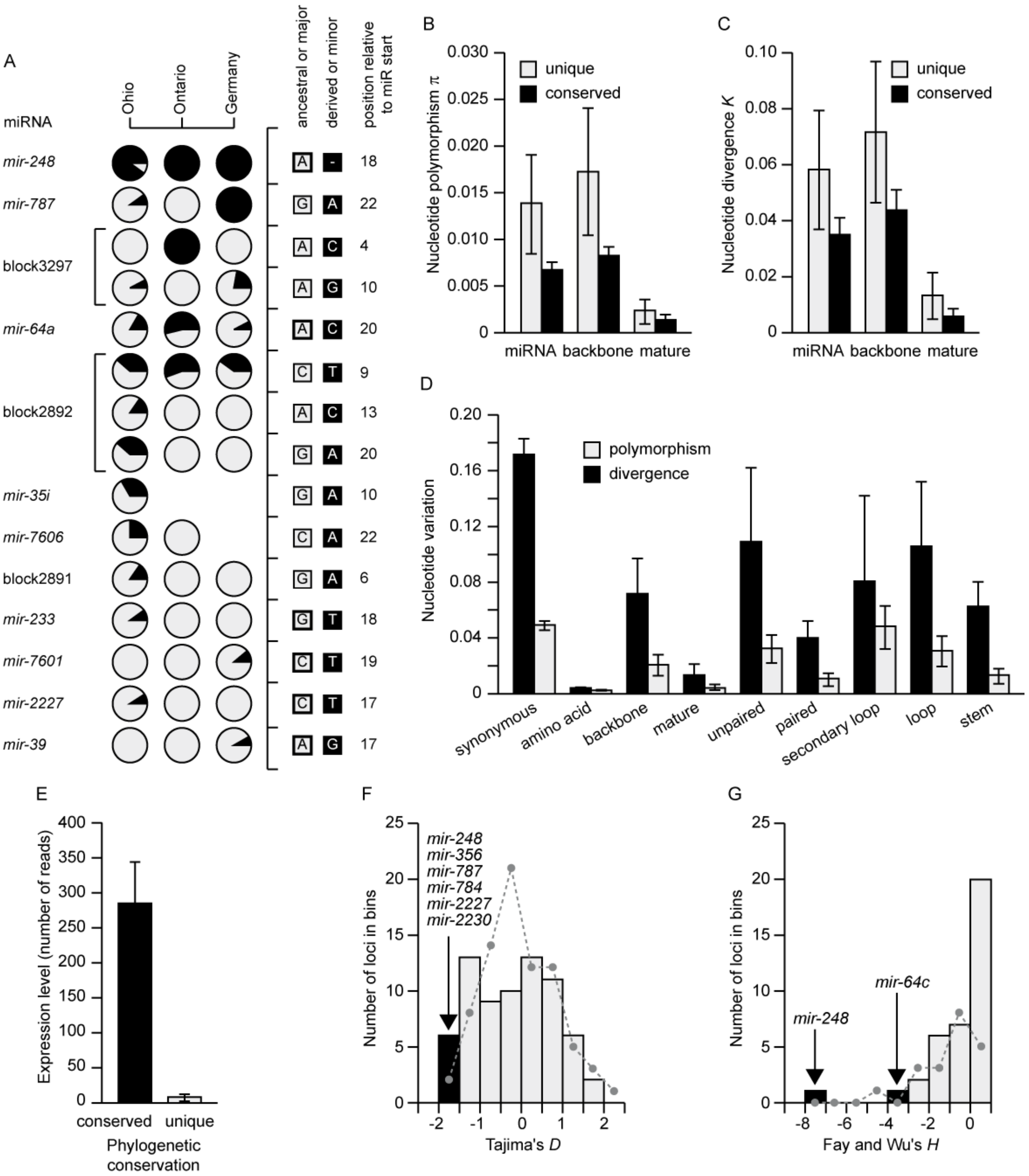

Supplementary Figure 8. Patterns of nucleotide variation after removing 21miRNAs with unusual amount of polymorphism are similar to the full miRNA dataset, showing that our general conclusions are robust to the sampling scheme. A. SNP frequencies in mature miRs among populations of *C. remanei*. Columns represent separate populations and rows represent distinct SNPs. Each circle represents the frequencies of the ancestral or major allele (in grey) and the derived or minor allele (in black). The different alleles and their position relative the start of

the mature miR are indicated in the rights panels. Ancestral alleles identified by comparison with *C. latens* are marked with a thick line. SNPs located in a same miRNA are joined by a horizontal bar. B, C. miRNAs from families unique to *C. remanei* and *C. latens* evolve faster than miRNAs from families conserved in other *Caenorhabditis* species. D. Nevertheless, young miRNAs show pervasive signatures of purifying selection. E. Young miRNAs are expressed at lower level than phylogenetically conserved miRNAs. F, G. Non-neutral pattern of sequence variation in miRNA hairpins. miRNAs with site frequency spectrum deviating from neutral expectations with Tajima's *D* (F) and Fay and Wu's *H* (G) are labeled in black, and miRNAs with SFS compatible with neutrality are labeled in grey. Note that these tests remain significant only for *mir-248* ($H < 0$) after correction for multiple-testing. The distributions of *D* and *H* for protein-coding genes are shown for comparison with dashed lines.

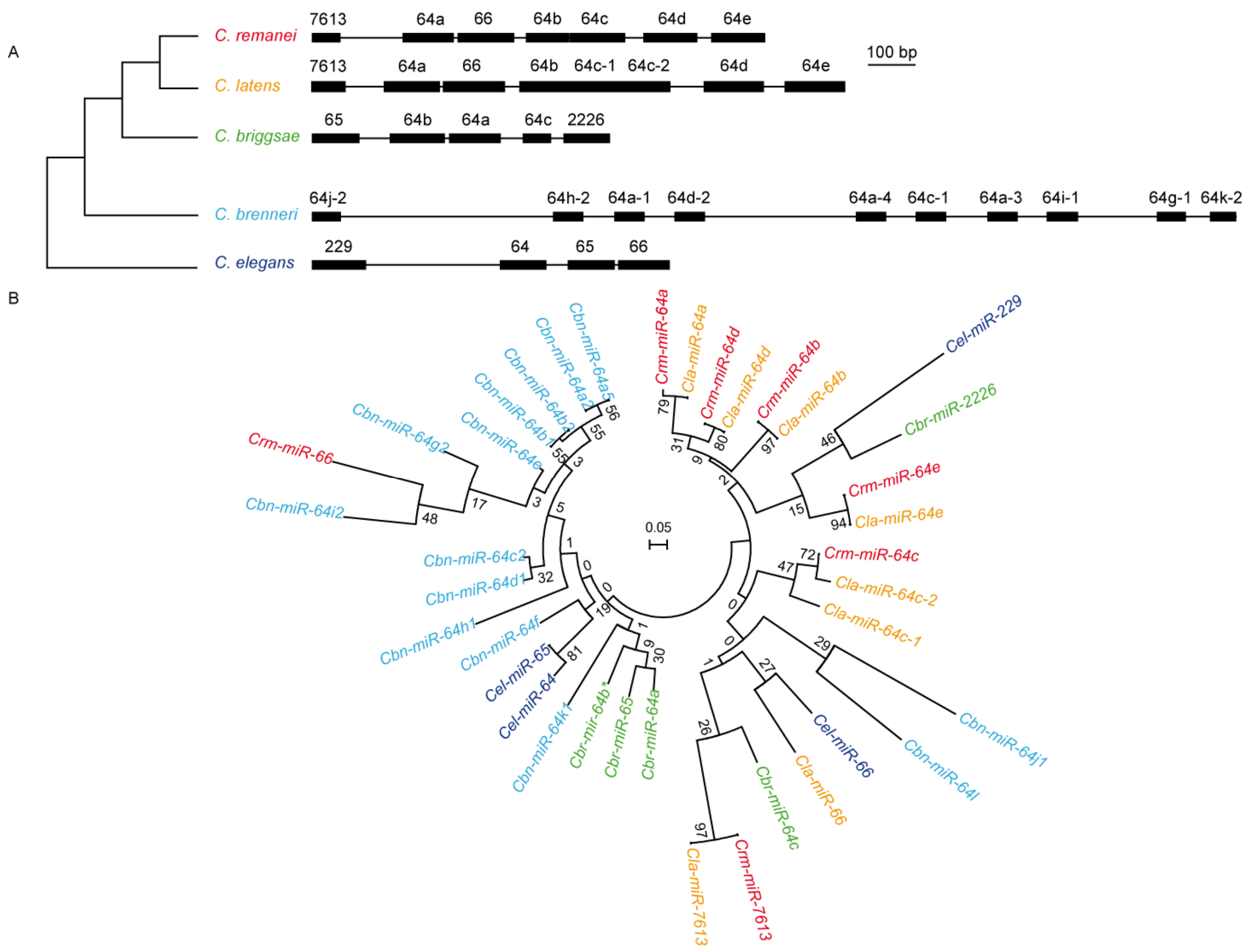

Supplementary Figure 9. A. The *mir-64* cluster has evolved by species-specific tandem duplications and/or miRNA losses. A. Structure of the *mir-64* cluster in five *Caenorhabditis* species. B. Phylogenetic relationships of the *mir-64* mature sequences. Numbers at each node indicate percent bootstrap support. *Crem: C. remanei; Cla: C. latens*

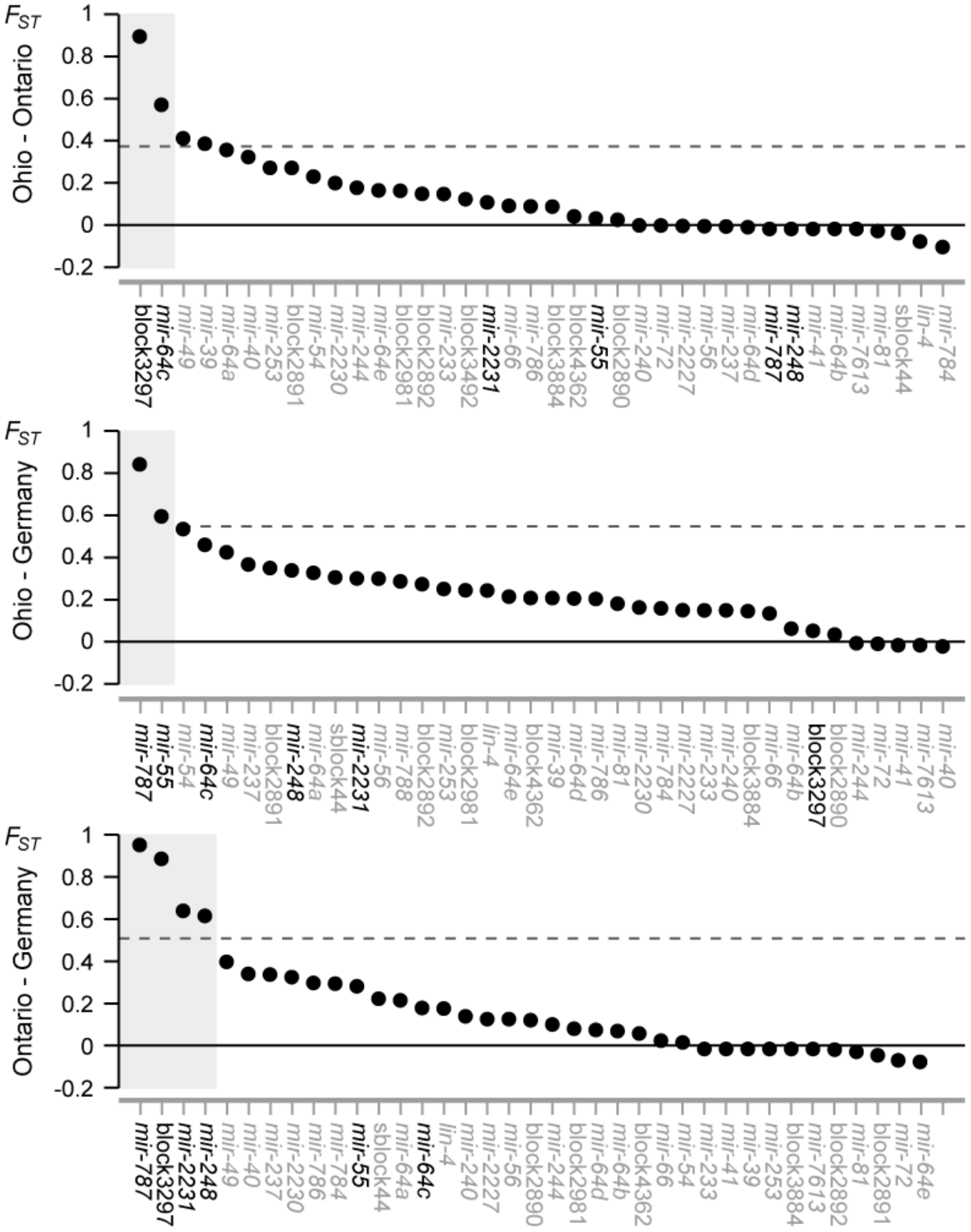

Supplementary Figure 10. Genetic differentiation of miRNAs between three populations of *C. remanei*. Each panel indicates the distribution of F_{ST} values between pairs of populations. Dots represent F_{ST} values measured with all polymorphisms located in the miRNA hairpin. The dashed lines represent 95th percentile of the empirical distribution of F_{ST} values in 20 re-sequenced protein-coding genes.

Supplementary Table 1. Summary statistics of nucleotide variation at miRNA loci in three populations of *C. remanei* and in *C. latens*.

		N[a]	n[b]	L[c]	P[d]	S[e]	π[f]
Flanking	OH[g]	209	10	183.4306	8.5024	1777	0.0179
	ON[h]	44	9	202.7955	7.4773	329	0.0211
	GER[i]	47	10	193.9787	6.6383	312	0.0219
	C. latens	97	2	190.4227	3.1031	301	0.0166
Hairpin	OH[g]	129	10	103.0310	2.9535	381	0.0095
	ON[h]	38	9	104.3421	3.9737	151	0.0144
	GER[i]	38	10	104.0526	4.0263	153	0.0150
	C. latens	65	2	103.6154	1	65	0.0079
Backbone	OH[g]	129	10	80.9690	2.8217	364	0.0115
	ON[h]	38	9	82.1842	3.8421	146	0.0177
	GER[i]	38	10	81.8421	3.8158	145	0.0181
	C. latens	65	2	81.1077	0.9538	62	0.0098
Mature	OH[g]	129	10	22.0620	0.1318	17	0.0020
	ON[h]	38	9	22.1579	0.1316	5	0.0024
	GER[i]	38	10	22.2110	0.2105	8	0.0033
	C. latens	65	2	22.5077	0.0462	3	0.0011
Star	OH[g]	39	10	21.6154	0.2308	9	0.0028
	ON[h]	15	9	21.7333	0.4	6	0.0062
	GER[i]	15	10	21.6667	0.5333	8	0.0082
	C. latens	28	2	21.75	0.0357	1	0.0005

[g]OH: Ohio; [h]ON: Ontario; [i]GER: Germany. [a]N: number of loci; [b]n: median number of strains; [c]L: average length; [d]P: average number of segregating sites; [e]S: total number of segregating sites per locus; [f]π: average nucleotide diversity per locus.

Supplementary Table 2. Selective constraints are stronger for amino-acid replacement sites in protein-coding genes than for mature miRNAs.

Sites	Within species nucleotide variation				Between species nucleotide variation			
	N[a]	π[b]	vs AA	vs Syn	N[a]	*K*[c]	vs AA	vs Syn
AA[d]	78	0.0015 ± 0.0002	NA	***	20	0.0036 ± 0.0008	NA	***
Syn[e]	78	0.0393 ± 0.0030	***	NA	20	0.1593 ± 0.0105	***	NA
flanking[f]	209	0.0179 ± 0.0011	***	***	133	0.0822 ± 0.0069	***	***
miRNA	129	0.0095 ± 0.0011	***	***	79	0.0380 ± 0.0052	***	***
backbone[g]	129	0.0115 ± 0.0014	***	***	79	0.0474 ± 0.0063	***	***
miR[h]	129	0.0020 ± 0.0006	***	***	79	0.0070 ± 0.0025	***	***
miR*[i]	39	0.0028 ± 0.0011	***	***	29	0.0032 ± 0.0022	***	***

Comparison of mean nucleotide polymorphism with the *C. remanei* population of Ohio and mean sequence divergence between nonsynonymous and synonymous sites with various site categories in miRNAs. Means are represented ± 1 standard error (SEM). [a]N: number of loci; [b]π: nucleotide diversity, [c]*K*: nucleotide divergence, [d]AA: amino acid replacement sites; [e]Syn: synonymous sites; [f]flanking: miRNA-flanking regions; [g]miR: mature sequence; [h]miR*: star sequence; NS: not significant; NA: not applicable. *** $P < 0.001$.

Supplementary Table 3. Comparison of nucleotide polymorphism in the *C. remanei* population of Ontario between nonsynonymous and synonymous sites with various miRNA site categories.

	Within species nucleotide variation			
Sites	N[a]	π[b] (mean ± SEM)	vs. AA	vs. Syn
AA[c]	19	0.0008 ± 0.0003	NA	***
Syn[d]	19	0.0368 ± 0.0063	***	NA
flanking[e]	44	0.0211 ± 0.0028	***	*
miRNA	38	0.0144 ± 0.0023	***	***
backbone[f]	38	0.0177 ± 0.0029	***	**
miR[g]	38	0.0024 ± 0.0014	***	***
miR*[h]	15	0.0062 ± 0.0031	NS	***

[a]N: number of loci; [b]π: nucleotide diversity; [c]AA: amino acid replacement sites; [d]Syn: synonymous sites; [e]flanking: miRNA-flanking regions; [f]miR: mature sequence; [g]backbone: miRNA minus miR; [h]miR*: star sequence; NS: not significant; NA: not applicable. * $P < 0.5$, ** $P < 0.01$, *** $P < 0.001$.

Supplementary Table 4. Comparison of nucleotide polymorphism in the *C. remanei* population of Germany between nonsynonymous and synonymous sites with various miRNA site categories.

	Within species nucleotide variation			
Sites	N[a]	π[b] (mean ± SEM)	vs AA	vs Syn
AA[c]	19	0.0006 ± 0.0002	NA	***
Syn[d]	19	0.0269 ± 0.0034	***	NA
flanking[e]	47	0.0219 ± 0.0035	***	NS
miRNA	38	0.0150 ± 0.0028	***	**
backbone[f]	38	0.0181 ± 0.0035	***	*
miR[g]	38	0.0033 ± 0.0017	*	***
miR*[h]	15	0.0082 ± 0.0036	NS	***

[a]N: number of loci; [b]π: nucleotide diversity; [c]AA: amino acid replacement sites; [d]Syn: synonymous sites; [e]flanking: miRNA-flanking regions; [f]miR: mature sequence; [g]backbone: miRNA minus miR; [h]miR*: star sequence; NS: not significant; NA: not applicable. * $P < 0.5$, ** $P < 0.01$, *** $P < 0.001$.

Supplementary Table 5. Homologs of miRNAs with signatures of adaptive selection in *C. remanei* regulate target genes involved in gonad formation in *C. elegans*. Only the cluster of functionally enriched genes with the highest score is shown for each miRNA.

miRNA	Cluster score	Enrichment	N	*P*	*P*-corrected
mir-787	5.23	sex differentiation	73	0.0000012	0.0011
		genitalia development	65	0.0000085	0.004
		reproductive development process	74	0.0000095	0.003
		hermaphrodite genitalia development	64	0.000013	0.003
mir-784	3.4	hermaphrodite genitalia development	54	0.00025	0.21
		genitalia development	54	0.0003	0.091
		reproductive development	61	0.00049	0.088
		sex differentiation	57	0.00067	0.086
mir-356	3.71	development of primary sexual characteristics	32	0.00012	0.042
		gonad development	30	0.00022	0.039
		reproductive structure development	30	0.00027	0.042
mir-248	3.93	reproductive developmental process	98	0.000033	0.018
		hermaphrodite genitalia development	83	0.000079	0.028
		genitalia development	83	0.0001	0.028
		sex differentiation	86	0.00072	0.076
mir-64	3.24	development of primary male sexual characteristics	6	0.0004	0.38
		male sex differentiation	6	0.0004	0.38
		male genitalia development	5	0.0012	0.21
mir-35	4.03	cell motion	13	0.000042	0.03
		cell migration	11	0.000071	0.025
		cell motility	11	0.00016	0.022
		localization of cell	11	0.00016	0.022

N: number of target genes, *P-corrected*: *P* value after Benjamini correction
